# *Porphyromonas gingivalis*-induced periodontitis promotes neuroinflammation and neuronal loss associated with dysfunction of the brain barrier

**DOI:** 10.3389/fcimb.2025.1559182

**Published:** 2025-06-09

**Authors:** Yiting Jiang, Lina Xu, Xuri Zhao, Hui Shen, Che Qiu, Zhiyan He, Zhongchen Song, Wei Zhou

**Affiliations:** ^1^ Department of Periodontology, Shanghai Ninth People’s Hospital, Shanghai Jiao Tong University School of Medicine, Shanghai, China; ^2^ College of Stomatology, Shanghai Jiao Tong University, Shanghai, China; ^3^ National Center for Stomatology, Shanghai, China; ^4^ National Clinical Research Center for Oral Diseases, Shanghai, China; ^5^ Shanghai Key Laboratory of Stomatology, Shanghai Research Institute of Stomatology, Shanghai, China; ^6^ Laboratory of Oral Microbiota and Systemic Disease, Shanghai Ninth People's Hospital, Shanghai Jiao Tong University School of Medicine, Shanghai, China; ^7^ Department of Stomatology, Taizhou Hospital of Zhejiang Province affiliated to Wenzhou Medical University, Zhejiang, China

**Keywords:** periodontitis, *P. gingivalis*, tau hyperphosphorylation, brain barrier, cognitive impairment

## Abstract

**Background:**

In our previous study, *Porphyromonas gingivalis* (*P. gingivalis*)-induced periodontitis caused cognitive impairment which was associated with abnormal amyloid β (Aβ) metabolite in the brain. The brain barrier is critical in maintaining homeostasis, controlling influx and efflux transport and regulating waste clearance. However, the specific role of the brain barrier in linking periodontitis to cognitive function remains unclear.

**Methods:**

A murine model of periodontitis-induced cognitive impairment was constructed via oral topical application of *P. gingivalis.* Neuroinflammation was observed by detecting the expression of proinflammatory cytokines and glia activation. Western blot (WB), immunohistochemistry (IHC) and immunofluorescence (IF) were used to detect the expression of tau-related molecules and neuronal loss. WB, Evans blue staining and flow cytometry were used to evaluate the blood-brain barrier (BBB) function including the infiltration of *P. gingivalis* and immune cells, and BBB permeability. The changes of meningeal lymphatic drainage were observed using an in vivo animal imaging system and reverse transcription polymerase chain reaction (RT-PCR). The effect of *P. gingivalis* on lymphatic endothelial cells (LECs) was further verified using IF and RT-PCR.

**Results:**

*P. gingivalis*-induced periodontitis exacerbated cognitive impairment by the upregulation of proinflammatory cytokine and glia activation. In the brain of periodontitis mice, p-Akt and p-GSK3β levels were reduced, leading to tau hyperphosphorylation and neuronal loss including cell bodies and neurites. *P. gingivalis*-induced periodontitis enhanced BBB permeability, promoted P. gingivalis and immune cell infiltration, and downregulated the expression of Occludin and ZO-1. In addition, the meningeal lymphatic drainage was impaired and the mRNA levels of lymphangiogenesis-related factor LYVE1 were decreased in the dura matter of periodontitis mice. After *P. gingivalis* infection, the inflammatory response was increased, and LYVE1 and ZO1 expression was decreased in LECs.

**Conclusions:**

Periodontitis aggravated neuroinflammation and neuronal loss which was associated with tau hyperphosphorylation. The impaired meningeal lymphatic vessels (MLV) and disrupted BBB affected the brain barrier function, further inhibiting the clearance of pathogenic substances and enhancing immune cell infiltration in periodontitis mice. These results indicated that brain barrier dysfunction may be the link between periodontitis and cognitive impairment.

## 1 Background

Periodontitis is a chronic inflammatory disease that occurs in periodontal supporting tissues and causes gingival bleeding, alveolar bone absorption and further tooth loss (Niu et al., 2021). As a driver of the chronic inflammatory response, periodontitis is closely related to systemic diseases such as neurodegenerative disease, cardiovascular disease, obesity, and adverse pregnancy outcomes (Liccardo et al., 2019; Hajishengallis and Chavakis, 2021; Pamuk and Kantarci, 2022; Xu and Han, 2022).

Evidence shows that periodontitis is a risk factor for Alzheimer’s disease (AD) patients (Dioguardi et al., 2020). The major neuropathological hallmarks of AD brains are amyloid beta (Aβ) and intracellular neurofibrillary tangles (NFTs). *P. gingivalis*, the keystone pathogen of periodontitis, is associated with inflammatory responses (Jia et al., 2019). *P. gingivalis* can release various virulence factors and toxins that enter the bloodstream, cause systemic inflammation, and access the brain via direct or indirect routes (Ding et al., 2018; Kanagasingam et al., 2020; Mulhall et al., 2020). Previous studies detected *P. gingivalis* in the brains of AD patients and mice by fluorescence in situ hybridization (FISH), quantitative polymerase chain reaction (qPCR) and nested qPCR (Singhrao et al., 2017; Dominy et al., 2019; Shen et al., 2024). Our earlier research suggested that periodontitis promoted neuroinflammation and caused cognitive impairment in rodents (Hu et al., 2020; Hu et al., 2021; Zhang X. et al., 2021). *P. gingivalis* invaded the brains of mice and led to abnormal amyloid precursor protein processing and Aβ metabolites (Shen et al., 2024). These previous studies have indicated that the abnormal influx and clearance of pathogens may be involved in cognitive impairment.

The brain barrier is important in maintaining homeostasis, controlling influx and efflux transport and regulating waste clearance in CNS. The glymphatic system and blood-brain barrier (BBB)—the main waste-clearance systems—are functionally linked with the influx and efflux of metabolic substances and cells. Louveau et al. (2015) was the first to discover functional lymphatic vessels, which carried both fluid and immune cells from the cerebrospinal fluid and were connected to the deep cervical lymph nodes (Da Mesquita et al., 2018a; Petrova and Koh, 2020). Aging is shown to be associated with the function of impaired meningeal lymphatic vessels (mLVs) (Guo et al., 2023). The disruption of mLVs in transgenic mouse models of AD can promote Aβ deposition in meninges (Da Mesquita et al., 2018b). However, the role of the brain barrier, especially mLVs, in periodontitis mice remains unknown.

The present study established a murine model of periodontitis via oral topical application of *P. gingivalis*. Behavioral tests were used to assess the learning and memory ability of the mice. Neuroinflammation was observed by detecting proinflammatory cytokine expression and glia activation. The tau-related molecules and neuronal loss were evaluated using western blot (WB), immunohistochemistry (IHC) and immunofluorescence (IF). WB, Evans blue staining and flow cytometry were used to evaluate BBB function including the infiltration of *P. gingivalis* and immune cells, and BBB permeability. The changes of meningeal lymphatic drainage were observed using an in vivo animal imaging system and reverse transcription polymerase chain reaction (RT-PCR).. Finally, IF and RT-PCR were employed to verify the effect of *P. gingivalis* on LECs.

## 2 Materials and methods

### 2.1 Establishment of periodontitis model

Forty-six eight-week-old male C57BL/6 mice were used from Vital River (Beijing, China). Mice were placed in a temperature-and humidity-controlled room under a 12-hour light/dark cycle, with free access to food and water. All experimental protocols were approved by the ethical committee of the Ethical Committee of Animal Care and Experimental Committee of the Ninth People’s Hospital, affiliated with Shanghai Jiao Tong University School of Medicine (SH9H-2019-A499-1).

*P. gingivalis* strain W83 was cultured anaerobically (85%N_2_,10%H_2_, 5%CO_2_) at 37°C. W83 was grown in Brain Heart Infusion Broth (OXOID, UK) supplemented with 1% hemin for 36~48 h. The concentration was determined using a spectrophotometer at an optical density of 600 nm.

Male mice were randomly divided into two groups: the control group (Control, n=23) and the experimental group (W83, n=23). Topical application of *P. gingivalis* at the buccal mucosa and gingiva of the maxillae three times a week was used to infect the mice in the W83 group (Papadopoulos et al., 2014). Mice in the control group were only administered 2% carboxymethyl cellulose (CMC). Each side was given 50 μL 2% CMC with/without *P. gingivalis* (1×10^10^ CFU/mL) locally.

**Figure 1:**
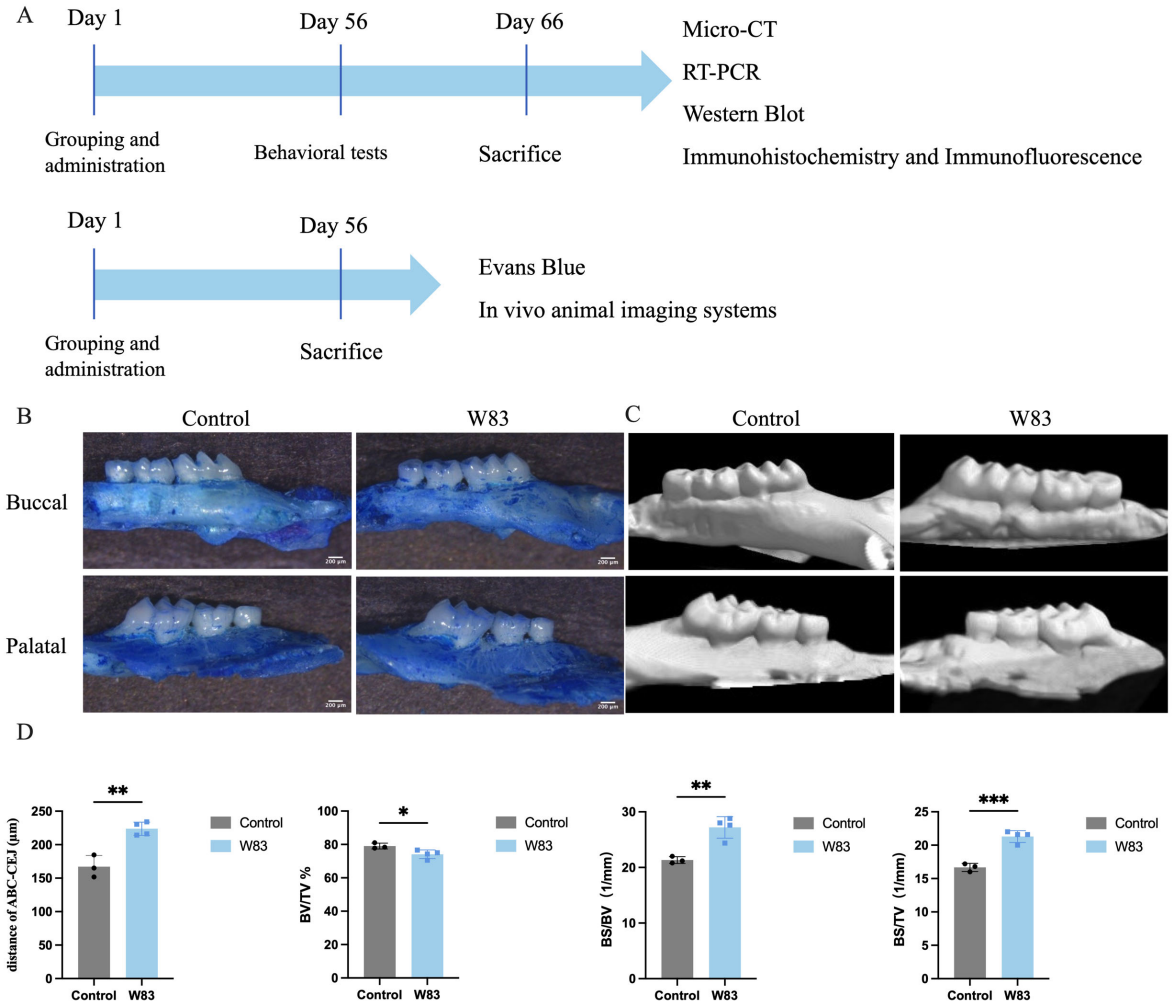
Experiment design *in vivo* and effects of oral topical application of *P. gingivalis* on alveolar bone. **(A)** *P. gingivalis* was administered to construct periodontitis mice model. After 8 weeks, locomotor activity and cognitive function were assessed by behavioral tests. Then the underlying mechanism was further detected by Micro-CT, RT-PCR, western blot, immunohistochemistry, flow cytometry, immunofluorescence, and *in vivo* animal imaging systems. **(B)** Images of maxillae by methylene blue staining. Scale bar=200μm. **(C)** Images of maxillae of microtomography. **(D)** Distance of ABC-CEJ, BV/TV, BS/BV, BS/TV. (*p<0.05, **p<0.01,***p<0.001, n=3-5 mice/ group).

After 8 weeks, some of the mice underwent behavioral testing (n=10/group) and were then euthanized. The gingiva, brain, spleen, and dura matter of the mice were used for further experiments. Methylene blue staining and microtomography of the maxillae were used to assess bone loss, as described below. In addition, the rest of the mice were used to evaluate BBB permeability (n=6/group) and lymphatic drainage (n=7/group). The *in vivo* study design is presented in **Figure 1A**.

### 2.2 Cell culture and *P. gingivalis* treatment

Lymphatic endothelial cells were purchased from the Anwei-Sci company (Shanghai, China). Cells were cultured in endothelial cell medium (Sciencell, USA) with 5% fetal bovine serum and 1% penicillin-streptomycin (100 μg/mL) at 37°C inside a 5% CO2 humidified incubator. The medium was replaced with a fresh, complete medium every three days. Cells were re-plated in 6- or 96-well cell culture microplates (3 × 10^5^ cells/well in 6-well microplates and 5× 10^3^ cells/well in 96-well microplates, Corning Life Sciences, USA). After the culture medium was replaced with fresh endothelial cell medium, lymphatic endothelial cells were infected with *P. gingivalis* (W83) at a multiplicity of infection of 50 (1:50) and 100 (1:100) for 24 h.

### 2.3 Measurement of alveolar bone resorption

The mice’s maxillae were fixed in 4% paraformaldehyde (PFA). The specimen was stained with 10 g/L methylene blue for 1 min. Photographs of the maxilla were obtained in the buccal and palatal aspects of the tooth. SkyScan 1272 (Bruker, Germany) was used to perform the alveolar bone morphometry. The morphometric parameters were measured using CT-Analyzer software. The region of interest (ROI) was standardized, extending from the cement-enamel junction (CEJ) to the root tip of the molar. Bone volume-to-tissue volume (BV/TV), bone surface-to-bone volume (BS/BV), and bone mineral density (BMD) were calculated.

### 2.4 Behavioral tests

#### 2.4.1 Open field test

The open field was a plastic box (40 cm×40 cm×40cm). Mice were placed in the corner of the box and were allowed to move freely for 5 min. Locomotor activity was captured by a fixed camera and processed using software (Noldus, Beijing, China).

#### 2.4.2 Novel object recognition

Two identical objects were placed in a plastic box (40 cm×40 cm×40 cm) on the first day. Mice were allowed to move freely for 5 min. The following day, one of the objects was replaced with a completely different object. Object exploration was defined as sniffing or touching the object with the nose or forepaws. The recognition index (the time spent exploring a novel object/total time spent exploring two objects) was used as an indicator of the mice’s memory of the object’s location.

#### 2.4.3 Y-maze

The Y-Maze involved three arms arranged at 120° angles. Mice were placed in the center of the experimental device and were allowed to explore for 5 min. Their movements were recorded with a camera positioned above the maze and were analyzed using a visual tracking system. The spontaneous alternation (%) =Alternations/(Arm entries - 2) ×100%. Alternations represented entry into a different arm from the previous two entries.

#### 2.4.4 Morris water maze test

The Morris water maze (MWM) test was conducted in a white circular pool with a diameter of 120 cm to test learning and memory abilities.

Over the course of four training days, mice were trained to find the platform hidden under the water within 90 s. If they did not find it, they were guided to stay on the platform for 15 s. Once mice could autonomously find and stay on the platform for 15 s, escape latency was recorded.

On the fifth day, the platform was removed. The mice were put into two quadrants of the pool to swim freely. The percentage of platform quadrant time and the number of platform crossings were recorded to evaluate recognitive function.

### 2.5 Reverse-transcription polymerase chain reaction

RNAiso Plus (Takara, Japan) was used to extract total RNA from the brain, gingival tissues and lymphatic endothelial cells according to the manufacturer’s instructions. Complementary DNA was synthesized using the Prime Script RT Master Mix (Takara, Japan). RNA extraction of dura matter was performed by using Total RNA Kit (Omega Bio-Tek, USA) and complementary DNA was synthesized by using Reverse Transcription Kit (Invitrogen, USA). The primer sequences of genes are shown in **Tables 1** and **2**. Each gene’s relative expression levels were then normalized to those of the housekeeping genes *β-actin* and *Gapdh* following the 2-ΔΔCt method (Wang et al., 2023).

**Table 1:** The primer sequences of genes for RT-PCR (Mice).

Gene (Mice)	Sequences
*β-actin*	Forward:5’-CATCCGTAAAGACCTCTATGCCAAC-3’
	Reverse:5’-ATGGAGCCACCGATCCACA-3’
*Il-1β*	Forward:5’- ACAACTGCACTACAGGCTCC-3’
	Reverse:5’- CTCTGCTTGTGAGGTGCTGA-3’
*Il-6*	Forward:5’- TAGTCCTTCCTACCCCAATTTCC -3’
	Reverse:5’- TTGGTCCTTAGCCACTCCTTC -3’
*Tnf-α*	Forward:5’-GACAAGGCTGCCCCGACTACG-3’
	Reverse:5’-CTTGGGGCAGGGGCTCTTGAC-3’
*Lyve1*	Forward:5’- GCAATGACATGTATGGCGGAG -3’ Reverse:5’- CTGTCCCAAGCAAGTGTGGA -3’
*Zo-1*	Forward:5’- ACCATGCCTAAAGCTGTCCC -3’ Reverse:5’- CCAACCGTCAGGAGTCATGG -3’
*Occludin*	Forward:5’- GCAATGACATGTATGGCGGAG -3’ Reverse:5’- CTGTCCCAAGCAAGTGTGGA -3’.

**Table 2:** The primer sequences of genes for RT-PCR (Human).

Gene (Human)	Sequences
*Gapdh*	Forward:5’- GAGAAGGCTGGGGCTCATTT -3’ Reverse:5’- AGTGATGGCATGGACTGTGG -3’
*Lyve1*	Forward:5’- CACTAGGCACCCAGTCCAAG -3’
	Reverse:5’- GTTGCGGGTGTTTGAGTGTC -3’
*Il-1β*	Forward:5’- ATGATGGCTTATTACAGTGGCAA-3’
	Reverse:5’- GTCGGAGATTCGTAGCTGGA-3’
*Tnf-α*	Forward:5’- CCTCTCTCTAATCAGCCCTCTG -3’
	Reverse:5’- GAGGACCTGGGAGTAGATGAG-3’

### 2.6 Enzyme-Linked Immunosorbent Assay (ELISA)

First, 40 mg of brain tissue was homogenized in 360 μL PBS. The samples were centrifuged at 12,000 rpm for 15 min at 4°C. Then, the supernatant medium was separated, immediately aliquoted into 1.5 mL cryogenic tubes, and frozen at −80°C until use. IL-1β (Neobioscience, China), IL-6 (Elabscience, China), and TNF-α (Shanghai Yingxin laboratory, China) levels were evaluated using ELISA kits according to the manufacturer’s instructions. A microplate reader was used to record the absorbance and OD values at 450 nm.

### 2.7 Immunofluorescence

Cells were fixed with 4% PFA and blocked with a blocking buffer (Beyotime, China) for 30 min at room temperature. Then, cells were incubated with primary antibodies (LYVE1, ZO1 1:200) overnight at 4°C. The following day, cells were incubated with Alexa Fluor 488/594 secondary antibodies (Invitrogen, 1:1000) for 1 h at room temperature. Then, cells were subjected to nuclear staining using DAPI (Beyotime, China). A DMi8 inverted microscope (Leica) was used to obtain images.

Half of the brain samples were fixed in 4% PFA at 4°C for 48 h. Next, they were dehydrated in 20% and 30% sucrose solutions for 24 h each. The brain tissues were embedded in OCT (SAKURA, Japan) and frozen for 10 min. A Leica frozen slicer (Leica CM1950 S, Leica, Germany) was used to cut sections at 20 μm thickness. Sections were blocked with a blocking buffer (Beyotime, China) for 30 min at room temperature and incubated with primary antibodies (β-III tubulin, NeuN 1:300) overnight at 4°C. Then sections were incubated with Alexa Fluor 488/594 secondary antibodies (Invitrogen, 1:1000) for 1 h at room temperature. After immunofluorescence staining, the sections were imaged in Z-stacks at an interval of 0.5 μm using a confocal laser microscope (Leica DMI8, Leica, Germany) with a 63× objective (oil). Imaris software was used to reconstruct 3D images.

### 2.8 Immunohistochemistry

Half of the brain samples were carefully removed and fixed in 4% PFA at 4°C for 48 h. Brains were dehydrated using an ethanol gradient and embedded in paraffin. After dewaxing and rehydrating the paraffin-embedded mouse brain tissues, sections were heated to 95°C for 20 min in an improved Citrate Antigen Retrieval Solution (Beyotime, China) and then naturally cooled to room temperature. A 3% hydrogen peroxide solution was applied for 10 min at room temperature. Sections were blocked with a blocking buffer (Beyotime, China) for 30 min at room temperature and incubated with primary antibodies (GFAP, Iba1, NeuN 1:200) overnight at 4°C.

The following day, sections were incubated with the corresponding HRP-labeled IgG polymer (Beyotime, 1:200), and counterstained with DAB (Beyotime, China) at room temperature. The sections were dyed with hematoxylin (Beyotime, China) for 5 min according to the manufacturer’s instructions and were dipped in 1% hydrochloric acid in alcohol for differentiation. Then, they were washed in the tap water for 10 min, and their nuclei became blue. A DMi8 inverted microscope (Leica) was used to obtain images. The density of GFAP or Iba1 positive cells was measured. The NeuN positive cells in the cortex and hippocampus were quantified using ImageJ (Huang et al., 2024).

### 2.9 Western Blot

First, 10 mg gingiva and 20 mg of brain tissues were homogenized in 90 μL and 180 μL RIPA lysis buffer supplemented with 1% PMSF and 2% protease and phosphatase inhibitor cocktail (Beyotime, China), respectively. Quantified proteins were mixed with 5 × sodium dodecyl sulfate (SDS) loading buffer and heated to 95°C for 5 min. Equal amounts of protein were separated using SDS polyacrylamide gel electrophoresis and transferred onto a PVDF membrane blocked with 5% skimmed milk as previously described (Li et al., 2018).

Then the membranes were incubated with anti-GAPDH (1:1000, Servicebio, China), anti-*P. gingivalis* (1:1000, MBL, Japan), anti-Akt (1:1000, Cell Signaling Technology, USA), anti-p-Akt (Ser473, 1:1000, Cell Signaling Technology, USA), anti-GSK3β (1:1000, Cell Signaling Technology, USA), anti-p-GSK3β (Ser9, 1:1000, Cell Signaling Technology, USA), anti Tau (1:1000, Cell Signaling Technology, USA), anti-p-Tau (S396, 1:1000, Cell Signaling Technology, USA), anti-p-Tau (S202, 1:1000, Cell Signaling Technology, USA), anti-ZO-1(1:3000, Proteintech, China), and anti-Occludin (1:1000, Proteintech, China) overnight at 4°C and incubated with HRP-linked Anti-Rabbit IgG (H + L) (1:1000, Cell Signaling Technology, USA) and Anti-Mouse IgG (H + L) (1:1000, Cell Signaling Technology, USA) for 1 h at room temperature. After 1 h, ECL Super (ThermoFisher Scientific, USA) was used to detect the protein bands, and the average densitometric analysis was processed using ImageJ.

### 2.10 Flow cytometry

Mice were anesthetized via intraperitoneal (i.p.) injection with 400 μL 1.25% tribromoethanol before being perfused with saline. The spleen, dura matter, and brain tissues were removed. The spleen was homogenized using a glass grinder in PBS. Red blood cell lysis buffer (Solarbio, China) was performed until the spleen cell pellets were no longer red. Brains were digested at 37°C for 50 min at 220 rpm with 100 μL 1 mg/mL collagenase IV in 1.9 mL PBS. Dura matters were digested with 200 μL 1 mg/mL collagenase IV in 500 μL PBS at 37°C for 20 min at 220 rpm. Then, PBS was added to terminate digestion. The digested solution was filtered through a 70 μm nylon-mesh filter. The single cell solution was centrifuged at 500 g for 5 min. Brain cell pellets were resuspended in 3 mL 30% Percoll (Cytiva, USA) and centrifuged at 800 g for 20 min without braking. The cell pellets were washed and resuspended in 100 μL PBS.

Cell viability was assessed by staining with the Zombie Aqua^™^Fixable Viability Kit, following the manufacturer’s instructions (BioLegend), and a single wash in PBS. Cells were incubated in 0.5 μL Fc Block (BD Pharmingen, USA) for 15 min at room temperature. Then, samples were stained for extracellular markers with the following antibodies for 30 min in the dark at 4°C: rat anti-11b PerCP-Cy5.5 (BD Pharmingen, USA), rat anti-CD45 APC-Cy7 (BD Pharmingen, USA), rat anti-Ly6G PE (BD Pharmingen, USA), rat anti-Ly6C FITC (BD Pharmingen, USA), and rat anti-B220 BV421 (Biolegend, USA). Cells were washed and fixed in 1% PFA. Data were collected using a flow cytometer (Beckman Coulter, Inc.) and then analyzed using FlowJo software (Tree Star, Inc.).

### 2.11 BBB permeability assay

BBB permeability was observed using Evans Blue staining at a dose of 8 mL/kg as previously described (Xue et al., 2022). After 1 h of circulation, mice were anaesthetized with tribromoethanol and perfused with saline. Their whole brains were carefully removed and photographed. Then, they were homogenized in 1 mL of 60% trichloroacetic acid solution and centrifuged at 14,000 g for 25 min. A supernatant was used to measure absorbance at 610 nm using a microplate reader (Epoch2, Bio-Tek, USA). The concentration was calculated via a standard curve. The BBB permeability index was calculated by dividing the value for each sample by the mean value of the control animals (Li et al., 2015).

### 2.12 *In vivo* animal imaging system

The ability of mLVs to drain biological molecules was evaluated by injecting Alexa Fluor 647-conjugated ovalbumin (OVA-647) into the intracisternal magna (i.c.m), as previously described (Li et al., 2022b). Mice were anaesthetized with tribromoethanol and placed in a stereotactic frame. Depilatory cream was applied to remove the hair on their necks. After making a 1-cm skin incision, the muscle layers were separated, and the atlanto-occipital membrane of the magna cisterna was revealed. OVA-647 (Thermo Fisher Scientific, USA) was injected into the cerebrospinal fluid-filled cisterna magna compartment slowly using a Hamilton syringe. To avoid leakage, the syringe was left in place for a few minutes after injection. After carefully closing the incision with a surgical suture carefully, the mice were placed on a heating pad until full recovery. Cervical images of live mice were captured using an *in vivo* animal imaging system at different time points (30 min, 60 min) after the administration of OVA-647 (IVIS, Lumina III, PerkinElmer, USA).

### 2.13 Statistical analysis

The normality of the data distribution was analyzed using the Shapiro-Wilk test. Normally distributed data were expressed as the mean ± standard deviation (SD). Unpaired *t*-tests were used to compare the two independent groups. Differences among the three groups were analyzed using one-way analysis of variance (ANOVA). *P* < 0.05 was considered statistically significant.

## 3 Results

### 3.1 Effects of oral topical application of *P. gingivalis* on alveolar bone

As shown in **Figures 1B** and **C**, methylene blue staining and Micro-CT were used to observe alveolar bone absorption. The distance of the cement-enamel junction–alveolar bone crest (CEJ-ABC) in the W83 group was significantly increased compared with the control group. As shown in **Figure 1D**, in the ROI of the W83 group, BV/TV was significantly lower than in the control group (79.06 ± 1.74% vs 74.1 ± 2.56%). BS/BV and BS/TV levels were significantly higher than in the control group (21.33±0.61/mm vs 27.20±1.93/mm, 16.67±0.61/mm vs 21.30±0.89/mm). These findings suggested infection with *P. gingivalis* in the oral cavity caused alveolar absorption.

### 3.2 Effects of *P. gingivalis* infection on locomotor activity and cognitive function

**Figure 2:**
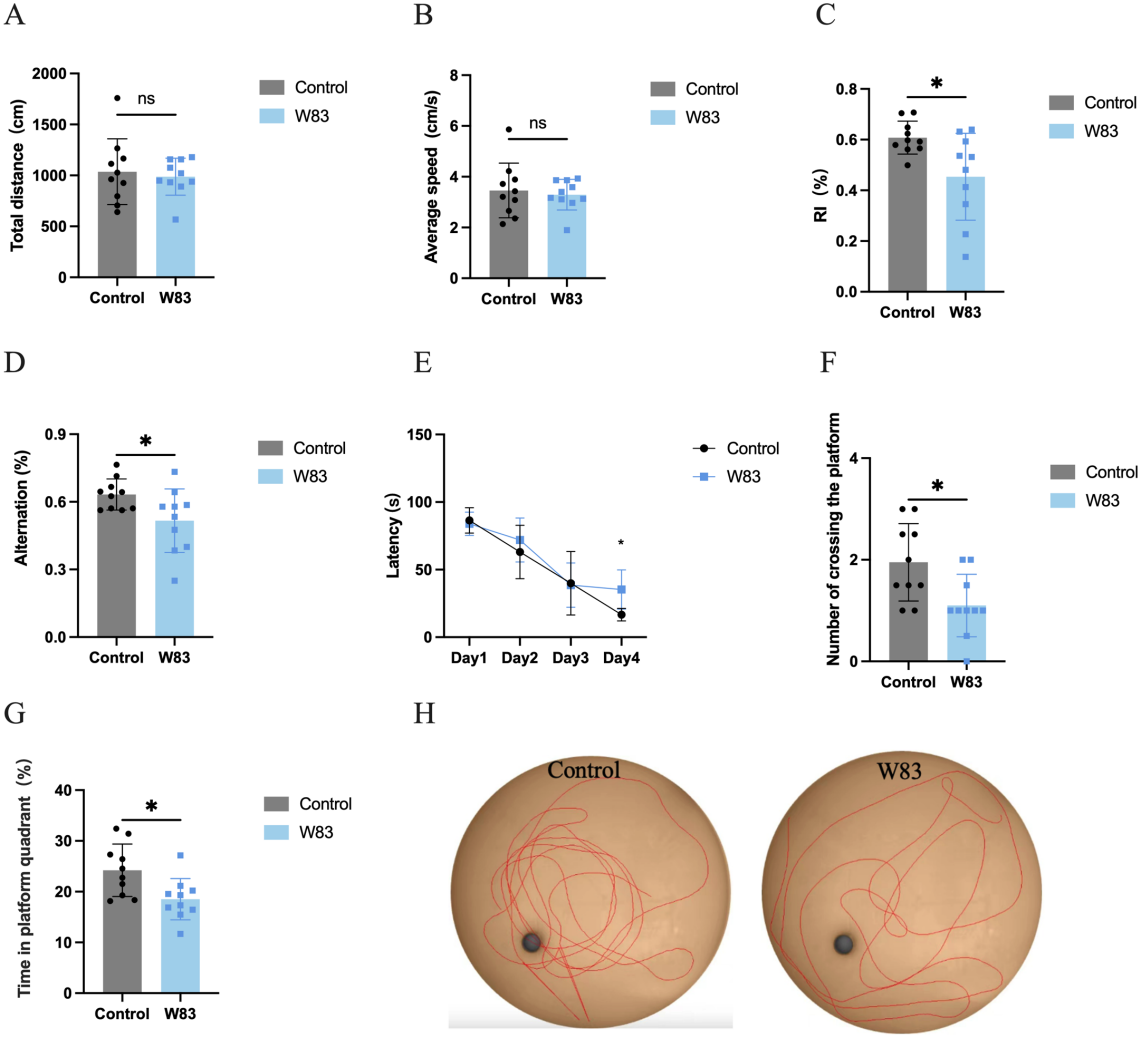
Effects of *P. gingivalis* infection on locomotor activity and cognitive function. The open field test was used to observe the locomotor activity of mice **(A, B)**. Novel object recognition **(C)** and Y maze **(D)** were used to evaluate the short-term memory. After these tests, MWM test was conducted to assess learning and memory ability (E~H). (*p<0.05, n=10).

As shown in **Figure 2**, the open field test (OFT) was used to evaluate the effects of periodontitis on locomotor activity. There was no significant difference in total distance traveled (**Figure 2A**) and average speed (**Figure 2B**) between the control and W83 groups.

Compared with mice in the control group, there was a marked decrease in the recognition index (**Figure 2C**) and alternation (**Figure 2D**) in the W83 group. This suggested that periodontitis induced by *P. gingivalis* impaired short-term memory.

The MWM test was used to explore the changes in learning and memory ability. There was a significant decrease in the latency in both groups over the four-day training period (**Figure 2E**). The latency was significantly longer in the W83 group than in the control group on the fourth day. On the fifth day, the platform was removed. The number of platform crossings and the percentage of platform quadrant time (**Figures 2F, G**) were significantly decreased in the periodontitis mice.. The tracks (**Figure 2H**) showed that the mice in the control group learned to navigate directly to the platform area even though it was hidden. These results indicated that *P. gingivalis*-induced periodontitis caused cognitive impairment.

### 3.3 Effects of *P. gingivalis* infection on neuroinflammation

**Figure 3:**
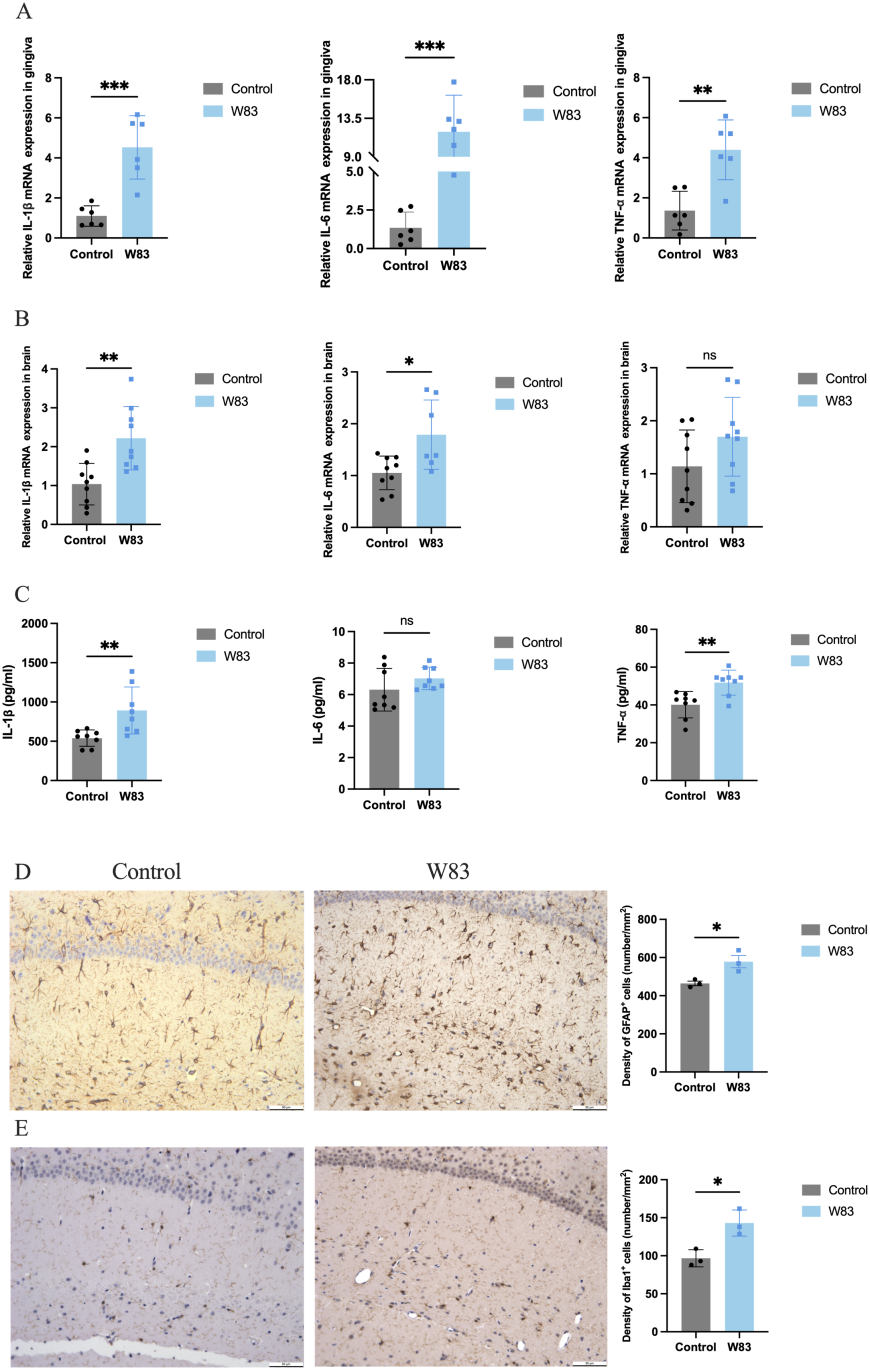
Effects of *P. gingivalis* infection on inflammation. **(A)** the mRNA expression of proinflammatory cytokines (*Il-1β*, *Il-6* and *Tnf-α*) was detected by RT-PCR in gingiva (n=6). **(B)** the mRNA expression of proinflammatory cytokines (*Il-1β*, *Il-6* and *Tnf-α*) was detected by RT-PCR in brain. **(C)** the protein expression of proinflammatory cytokines (*Il-1β*, *Il-6* and *Tnf-α*) was detected by ELISA in brain (n=8). **(D)** Representative images and number of GFAP positive cells in brain. **(E)** Representative images and number of Iba1 positive cells in brain. (Scale bar=50 µm, *p<0.05, **p<0.01, ***p<0.001).

The mRNA levels of proinflammatory cytokines *Il-1β*, *Il-6* and *Tnf-α* were increased significantly in the gingiva and brain in the W83 group (**Figures 3A, B**). IL-1β, IL-6, and TNF-α protein levels in the brain were increased in the W83 group (**Figure 3C**). Further, the activation of astrocytes and microglia in the brain was clearly observed in the W83 group. The number of positive cells was increased in the brain of periodontitis mice (**Figures 3D, E**). These results demonstrated that *P. gingivalis* infection promoted local inflammation in periodontal tissues and further increased inflammatory levels in the CNS. *P. gingivalis*-induced periodontitis may promote neuroinflammation.

### 3.4 Effects of *P. gingivalis* infection on tau hyperphosphorylation and neurons

**Figure 4:**
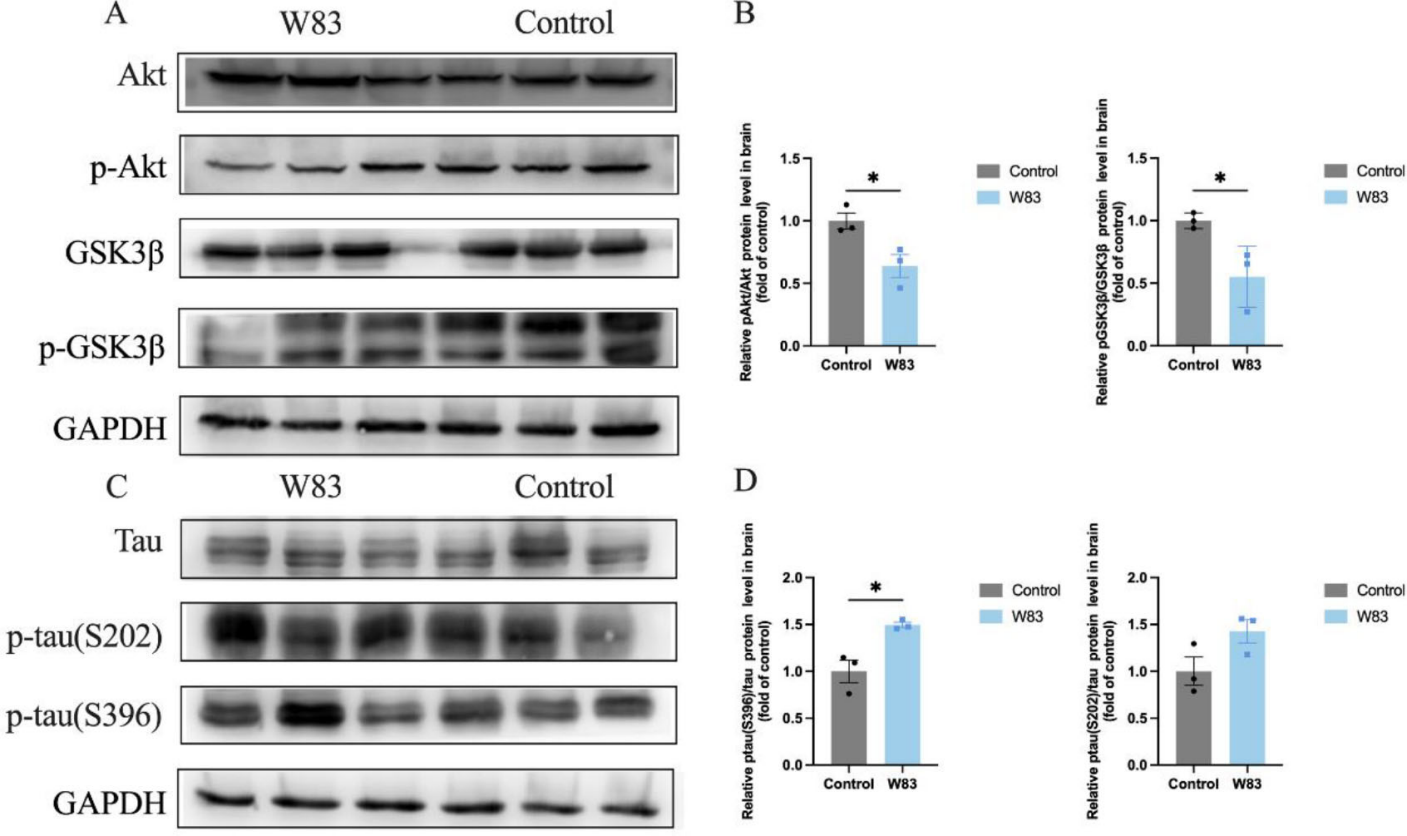
Effects of *P. gingivalis* infection on phosphorylation of tau protein in brain. (**A–D)**, Western Blot was performed to evaluate the level of Akt, p-Akt, GSK3β, p-GSK3β protein in brain (n=3). **(A)** Representative bands for Akt, p-Akt, GSK3β, p-GSK3β and GAPDH in gingiva. **(B)** Protein expression levels were normalized with the corresponding GAPDH level. **(C)** Representative bands for Tau, p-tau and GAPDH in brain (n=3). **(D)** Protein expression levels were normalized with the corresponding GAPDH level. (*p<0.05).

Glycogen synthase kinase-3β (GSK-3β) is a major kinase responsible for tau hyperphosphorylation in AD. Western blot was used to evaluate Akt and GSK-3β levels in the brain. As shown in **Figures 4A** and **B**, p-Akt and p-GSK-3β were decreased in the brains of periodontitis mice compared with the control group. The protein expression level of phosphorylated tau at the Ser396 site was significantly upregulated in the brains of periodontitis mice (**Figures 4C, D**).

**Figure 5:**
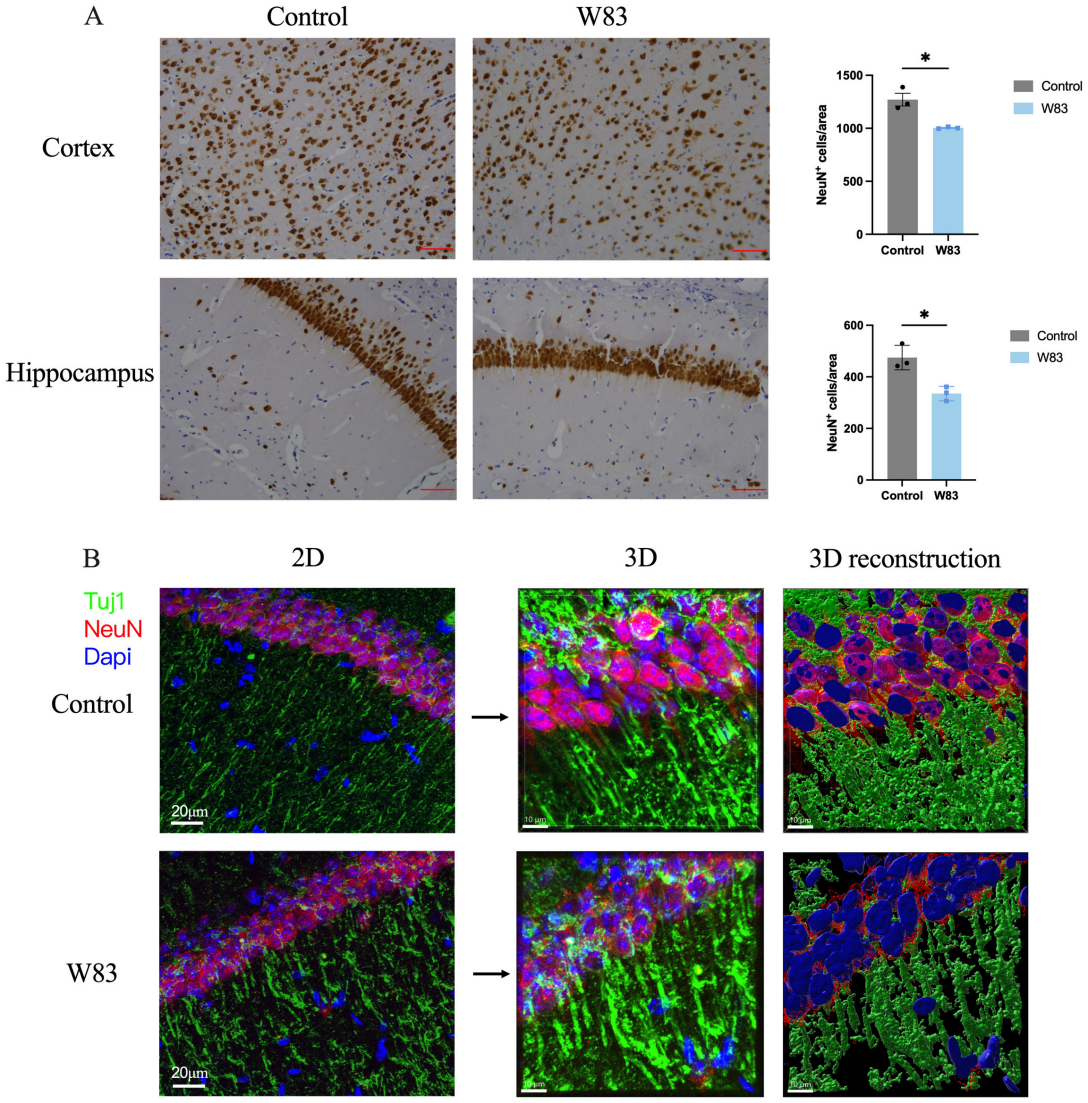
Effects of *P. gingivalis* infection on neurons in brain. **(A)** Representative pictures and number of NeuN positive cells in cortex and hippocampus. (Scale bar =25 μm, * p<0.05, n=3). **(B)** Representative images of immunofluorescence staining for Tuj1 (green), NeuN (red) and DAPI (blue) in CA1. (Scale bar=20 μm) 3D schematic diagram of immunofluorescence staining observed using confocal microscopy (Scale bar =10 μm).

As shown in **Figure 5A**, NeuN positive neurons in the brain of periodontitis mice were decreased significantly. The cell bodies (labeled with NeuN) and neurites (labeled with Tuj1) of neuron were observed by immunofluorescence staining and 3D reconstruction technology (**Figure 5B**). We observed that the density of Tuj1 in W83 group was decreased, which may be due to the decline of neuronal cell bodies. Compared with the Control group, neurites of neurons in the W83 group were more discontinuous.

These results demonstrated that periodontitis may enhance tau hyperphosphorylation via the Akt/GSK-3β pathway, affecting neurons in the cortex.

### 3.5 Effects of *P. gingivalis* Infection on blood-brain barrier

**Figure 6:**
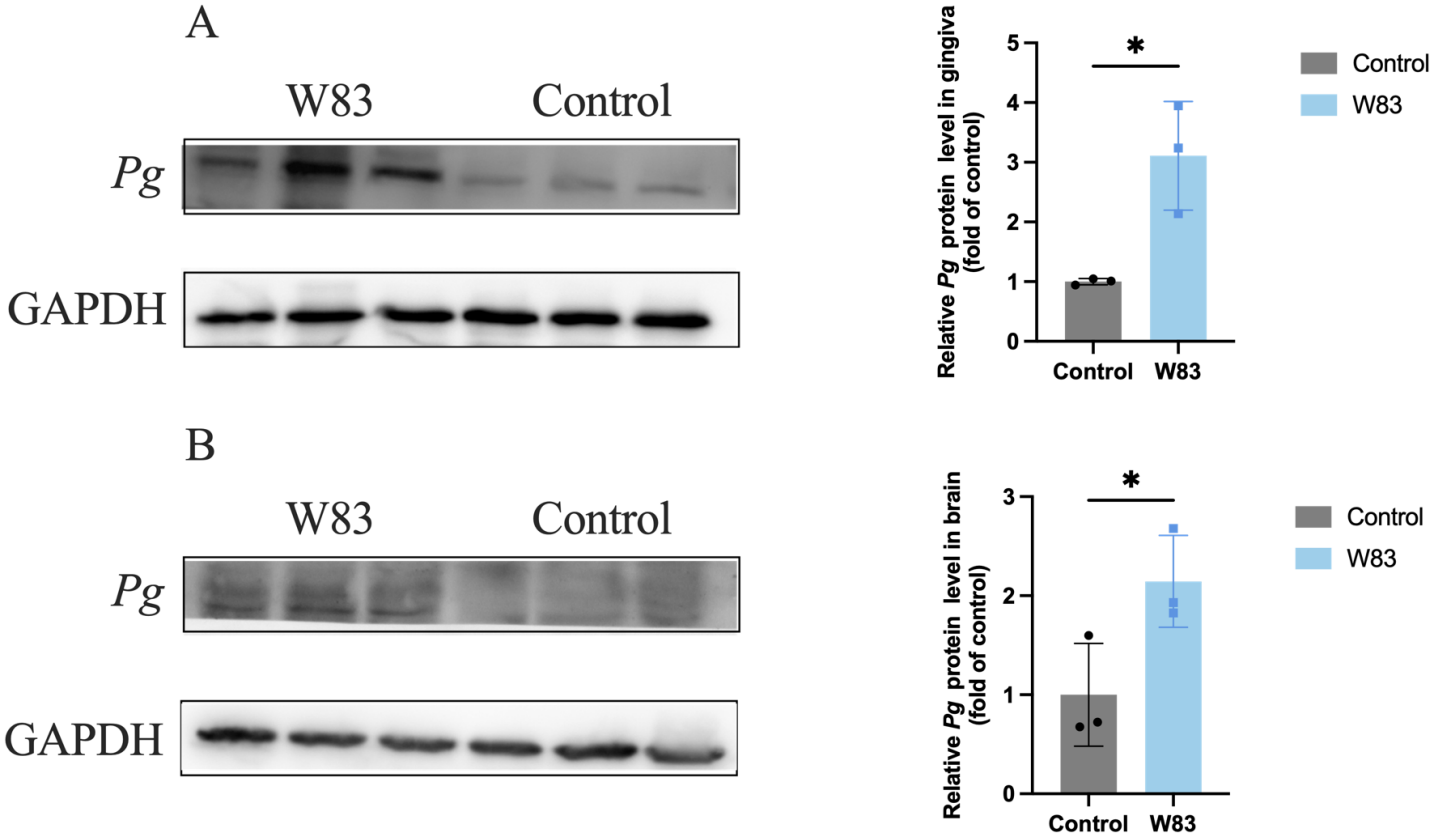
Detection of *P. gingivalis* in gingiva and brain. Western Blot was performed to detect *P. gingivalis* in the gingiva and brain (n=3). **(A)** Representative bands for *P. gingivalis* and GAPDH in gingiva. The level of *P. gingivalis* protein in gingiva was normalized with the corresponding GAPDH level. **(B)** Representative bands for *P. gingivalis* and GAPDH in brain. The levels of *P. gingivalis* protein in brain was normalized with the corresponding GAPDH level. (*p<0.05).

Western Blot was used to detect *P. gingivalis* in the gingiva (**Figure 6A**) and brain (**Figure 6B**). The level of *P. gingivalis* in the gingiva was increased in the W83 group. Additionally, the detection of *P. gingivalis* in the brain showed similar results. These findings revealed that *P. gingivalis* could colonize the gingiva and invade the brains of mice.

**Figure 7:**
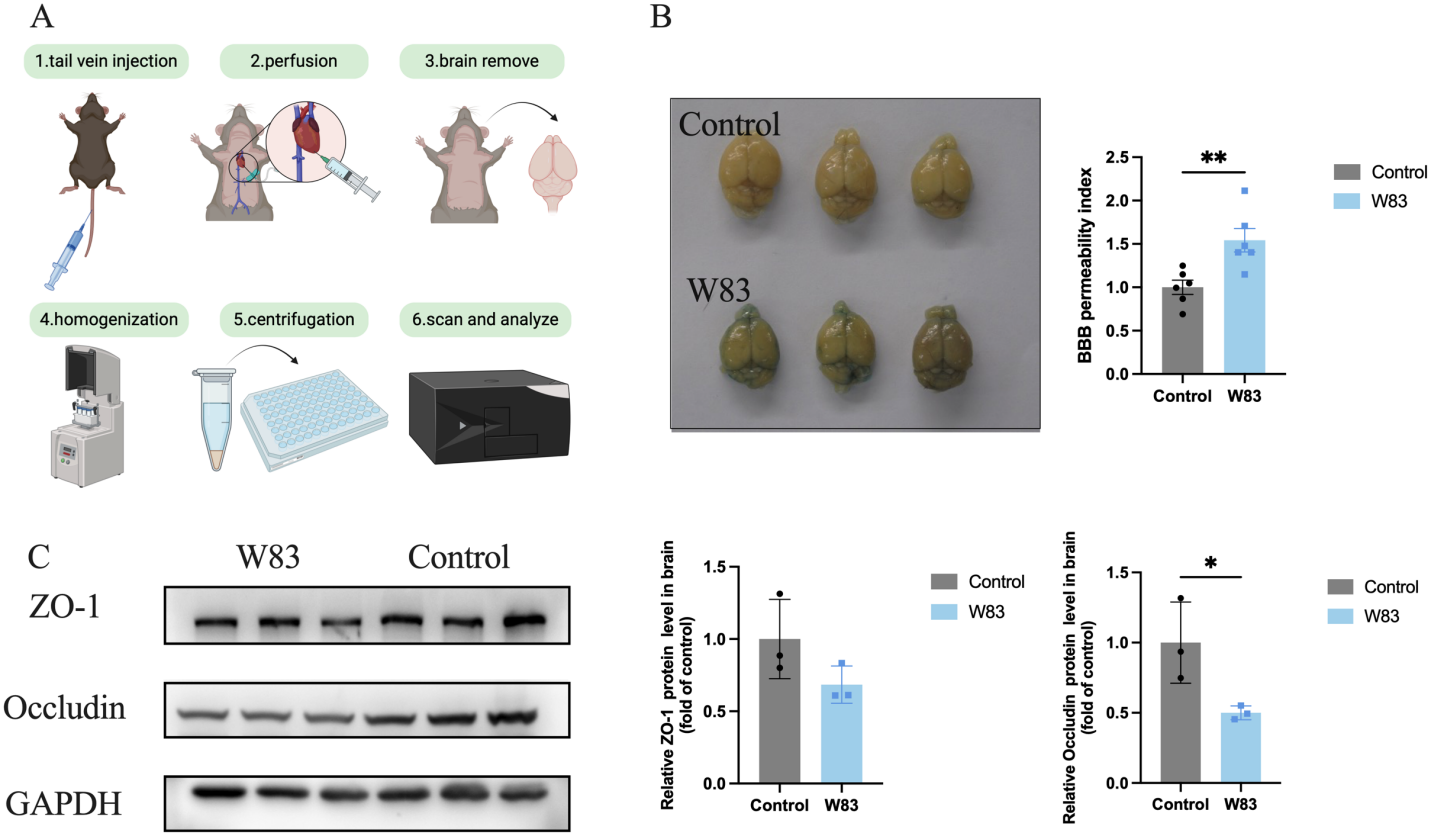
Effect of *P. gingivalis* infection on blood-brain barrier. **(A)** Injecting 2% Evans Blue staining via tail vein, after 1h, the mice were perfused with saline. Then the brains in both groups were photographed. The amount of Evans Blue was measured. **(B)** The representative pictures of brain in both groups and BBB permeability index. **(C)** Representative bands for ZO-1, Occludin and GAPDH in brain. The level of ZO-1 and Occludin protein in brain was normalized with the corresponding GAPDH level. (*p<0.05, **p<0.01).

The BBB permeability assay was shown in **Figure 7A**. The The BBB permeability index increased significantly in the W83 group (**Figure 7B**). Compared with the control group, the expression of Occludin and ZO-1 protein was decreased in the brain of periodontitis mice (**Figure 7C**), indicating that *P. gingivalis* infection led to BBB disruption.

**Figure 8:**
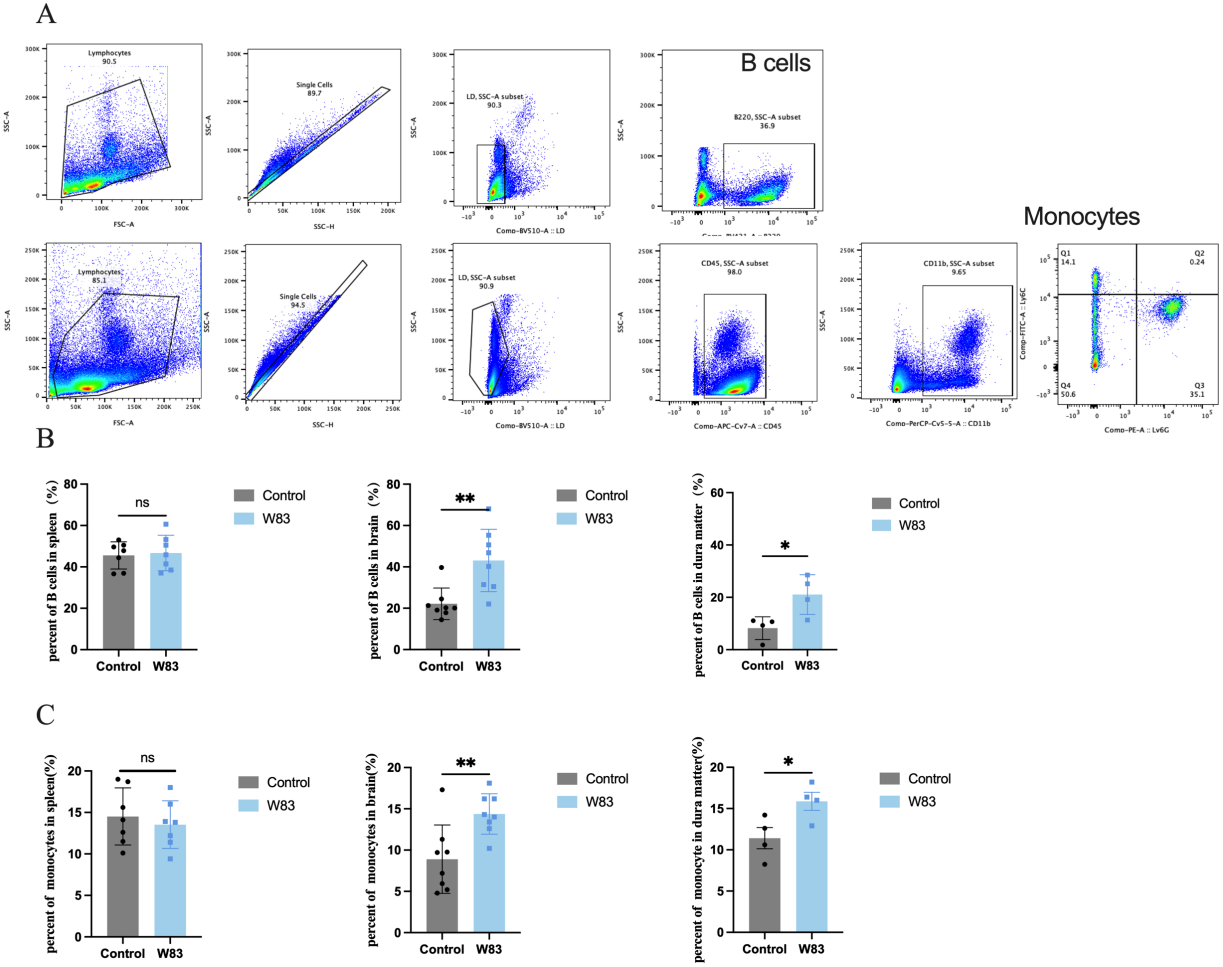
Effects of *P. gingivalis* infection on immune cells percentage. **(A)** Flow cytometric gating strategy of immune cells including B cells (B220^+^) and monocytes (CD45^+^CD11b^+^Ly6C^+^Ly6G^-^). **(B)** Percentage of B cells in spleen, brain and dura matter. **(C)** Percentage of monocytes in spleen, brain and dura matter. (*p<0.05, **p<0.01, n=4-8mice/group).

We investigated whether *P. gingivalis* altered immune cell infiltration in the peripheral tissue and CNS. Single-cell suspensions extracted from the spleen, brain, and dura matter were analyzed using flow cytometry. The gating strategy is shown in **Figure 8A**. As illustrated in **Figures 8B** and **C**, there were no significant differences in fractions of B cells (B220^+^) and monocytes (CD45^+^CD11b^+^Ly6C^+^Ly6G^-^) in the spleen between the two groups. However, compared with the control group, the proportions of B cells and monocytes in the brain and dura mater increased in the W83 group (**Figures 8B, C**).

These above results showed that periodontitis mice led to BBB disruption followed by the infiltration of *P. gingivalis* and immune cell, and the increase of BBB permeability.

### 3.6 Effects of *P. gingivalis* Infection on meningeal lymphatic system

**Figure 9:**
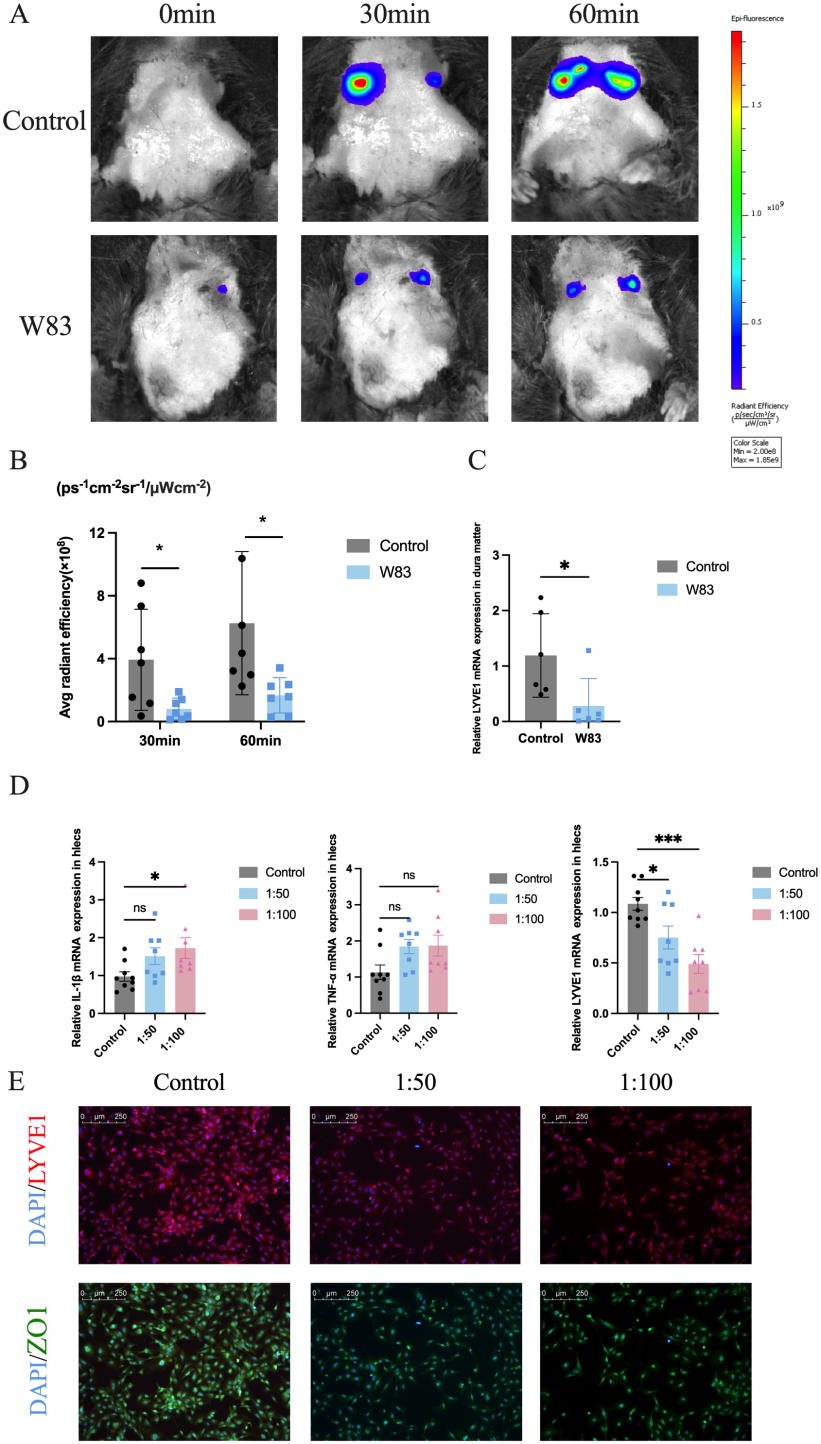
Effects of *P. gingivalis* infection on meningeal lymphatic system *in vivo* and lymphatic endothelia cells *in vitro.* **(A)** Images of live mice in both groups were captured at different time points after administration of OVA-647 using an *in vivo* animal imaging system. **(B)** Fluorescence accumulation of OVA-647 in the cervical region was quantified (n=7). **(C)** Relative *Lyve1* mRNA expression in dura matter. (n=6). **(D)** Relative mRNA expression levels of *Il-1β*, *Tnf-α* and *Lyve1* in lymphatic endothelial cells which were infected by *P. gingivalis* for 24h (n=7-9 samples/group). **(E)** Representative LYVE1 and ZO1 staining by IF. Scale bar=250μm. (*p<0.05, ***p<0.001).

To further evaluate mLVs’ ability to drain biological molecules, we performed fluorescence imaging *in vivo* to monitor the kinetic biodistribution of Alexa Fluor 647-conjugated ovalbumin (OVA-647) via the intracisternal magna (i.c.m). Images were captured from the ventral view of mice at various time points up to 1h after administration. Fluorescent signals could be detected in the cervical regions of the mice, confirming that the fluorescent signals were derived from cervical lymphatic nodes (CLNs), but not from other local tissues. The intensities of OVA-647 fluorescent signals in cervical regions (**Figures 9A, B**) were significantly decreased in periodontitis mice at 30 min or 60 min after administration of OVA-647. As shown in **Figure 9C**, LYVE1 expression levels were decreased significantly in the dura matter of periodontitis mice.

In addition, we used lymphatic endothelial cells as an *in vitro* model to study the effect of *P. gingivalis* infection. As shown in **Figure 9D**, the mRNA expression levels of proinflammatory cytokines *Il-1β* and *Tnf-α* were increased significantly after *P. gingivalis* infection for 24 h. The mRNA levels of mLVs marker *Lyve1* were decreased after *P. gingivalis* stimulation. Immunofluorescence staining was performed to evaluate the effect of *P. gingivalis* on lymphangiogenesis-related factor (LYVE1) and tight junction protein (ZO1). As shown in **Figure 9E**, the fluorescence intensity of LYVE1 and ZO1 was downregulated after *P. gingivalis* infection compared with the control group.

The above findings indicated that *P. gingivalis*-induced periodontitis could promote inflammatory response and decrease the expression of ZO-1 and LYVE1, leading to impaired meningeal lymphatic drainage.

## 4 Discussion

In this study, periodontitis was induced by oral topical application of *P. gingivalis*. Periodontitis aggravated neuroinflammation and neuronal loss, which was associated with tau hyperphosphorylation. The disruption of BBB in periodontitis mice caused pathogens and immune cell infiltration. Impaired meningeal lymphatic drainage inhibited metabolic substances clearance. These results indicated that brain barrier dysfunction, especially the mLV, was the potential target connecting periodontitis and cognitive impairment.

*P. gingivalis*, the key pathogen of periodontitis releases various virulence factors (Xu et al., 2020; Zhang Z. et al., 2021). Considering the trauma of ligature model (Marchesan et al., 2018), we induced periodontitis mice using oral *P. gingivalis* infection (Kang et al., 2022; Xiao et al., 2022). *P. gingivalis* W83, a whole-genome sequenced bacterium, was isolated from a clinical specimen. Evidence has shown that W83 is more virulent than ATCC33277 (Werheim et al., 2020; Xie et al., 2025). Micro-CT and methylene blue staining showed, that the increased distance of CEJ-ABC in the W83 group could be observed, confirming the success of the periodontitis model.

Periodontitis is one of the high-risk factors for AD patients (Pazos et al., 2018; Beydoun et al., 2020; Guo et al., 2021; Asher et al., 2022). The MWM, NOR, and Y maze are commonly used behavioral methods (Vorhees and Williams, 2006; Lueptow, 2017; Shi et al., 2021). Previous studies have found that periodontitis mice induced by *P. gingivalis* (ATCC33277) may exacerbate brain Aβ deposition and caused cognitive impairments (Ishida et al., 2017; Shen et al., 2024). In our study, periodontitis induced by *P. gingivalis* (W83) could cause cognitive impairment.

Periodontitis could cause systemic inflammation and further promoted the neuroinflammation (Wang RP. et al., 2019; Li et al., 2022a). Pathogens or debris from damaged cells may activate the resting microglia and astrocytes to express IL-1β and TNF-α (Kwon and Koh, 2020). Previous studies have shown that the levels of proinflammatory cytokines were increased in the brain of periodontitis mice infected with *P. gingivalis* (ATCC33277) (Zhang et al., 2018; Cao et al., 2024). In our study, more activated microglia and astrocytes were observed in periodontitis mice. *Il-1β*, *Il-6*, and *Tnf-α* levels were increased significantly in the gingiva and cortex in periodontitis mice, which was similar to the results of previous studies (Hu et al., 2020; Li et al., 2023).

Gram-negative bacteria can themselves trigger neuronal cell death as well as through their byproducts (Kim et al., 2021). An *in vitro* study also indicated that *P. gingivalis* could invade and lead to neuronal damage (Haditsch et al., 2020). *Pg-*OMV promotes tau phosphorylation, synapse loss, and disordered neuron arrangement (Gong et al., 2022; Jiang et al., 2025). In recent years, researchers have found that GSK3β overactivation may promote tau phosphorylation and regulate neuronal functions in CNS (Lauretti et al., 2020; Lee et al., 2024; Zhao et al., 2024). *Pg*-LPS induced tau hyperphosphorylation at different sites in the neurons of mice (Jiang et al., 2021). In our study, periodontitis promoted tau hyperphosphorylation at the S396 site. The NeuN-positive cells in the brains of periodontitis mice were also decreased. These results indicated that periodontitis increased tau hyperphosphorylation and caused neuronal loss by modulating the Akt/GSK3β pathway.

Brain barriers are important for maintaining homeostasis and complex functions. The BBB is a key structural and functional barrier with low permeability. BBB disruption can be observed in AD brains (Wang et al., 2022) and allows influx into the brain of cells and microbial pathogens (Sweeney et al., 2018; Peng et al., 2021). *P. gingivalis* and its virulence factors, such as Pg-OMV, LPS, and gingipain, could induce chronic inflammation and damage BBB function (Nonaka et al., 2022; Pritchard et al., 2022; Lei et al., 2023; Jiang et al., 2025). Li et al. (2024) also found that gingipains may impair cognition function and enhance BBB permeability *in vivo* and *in vitro*. Consistent with the above findings, we found that *P. gingivalis* infection enhanced BBB permeability, which may allow *P. gingivalis* to enter the CNS.

LPS and gingipain have been detected respectively in the brain tissues of AD patients (Poole et al., 2013; Dominy et al., 2019). The presence of *P. gingivalis* was confirmed in rodents’ brains by PCR, FISH and qPCR (Poole et al., 2015; Singhrao et al., 2017; Díaz-Zúñiga et al., 2020). In the present research, we use Western blot to detect *P. gingivalis* in the brain. Previous research has confirmed that this anti-*P. gingivalis* antibody does not cross-react with other bacterial species, indicating its specificity. LPS may be the target antigens of this specific antibody (Rajakaruna et al., 2018). In our previous study, we also detected *P. gingivalis* DNA in brain tissues using nested quantitative PCR (Shen et al., 2024). The above results revealed that *P. gingivalis* DNA and specific proteins could be detected in *P. gingivalis*-induced periodontitis mice.

Neuroimmune interactions are crucial for regulating immunity and inflammation responses. Circulating monocytes infiltrated the CNS through a weakened or impaired BBB (Quarta et al., 2021). Park et al. (2022). have found that a longitudinal increase in B lymphocytes is associated with increased cerebral amyloid deposition in AD brains. In our study, the percentage of monocytes and B cells was increased in the brain and dura matter in periodontitis mice, indicating that oral infection with *P. gingivalis* could promote immune cell infiltration into the CNS.

Improving lymphatic drainage could promote the clearance of misfolded, disease-related proteins including Aβ and tau, in different mice models (Patel et al., 2019; Da Mesquita et al., 2021). mLVs are composed of cells that express the cardinal marker for lymphatic endothelium-the lymphatic vessel hyaluronan receptor 1 (LYVE1) (Brezovakova and Jadhav, 2020). Wang et al (Wang et al., 2024). found that lymphatic drainage and cognitive function were impaired in 5xFAD mice. Blocking mLVs drainage exacerbates AD-related pathology in APP/PS1 mice (Wang L. et al., 2019). We found that periodontitis impaired mLV function and downregulated LYVE1 expression. This result may be related to AD-associated pathology by reducing tau clearance, finally contributing to cognitive impairment. Additionally, *P. gingivalis* infection caused obvious inflammation activation and a decrease of lymphatic markers such as LYVE1 in an *in vitro* model established by lymphatic endothelial cells.

The anatomical relation and the constant swallowing of oral bacteria make oral-gut communication possible. Pathogens may also cross a weakened oral barrier to the vascular circulation (Panza et al., 2019). In our previous study, *P. gingivalis* was detected in the blood and cortex of periodontitis mice (Shen et al., 2024). In this research, *P. gingivalis* was detected in the gingiva and brain. The exact mechanism needs further study, such as oral-gut axis.

In this work, the periodontitis model which was induced by *P. gingivalis* leaded to learning and memory impairment. We found that periodontal inflammation could promote neuroinflammation and tau hyperphosphorylation, leading to neuronal loss. Periodontitis could also cause increased BBB permeability and impaired mLV drainage, affecting the clearance of pathogens and tau protein. These findings suggest that the brain barrier dysfunction is a potential target for connecting periodontitis with cognitive impairment.

## 5 Conclusions

*P. gingivalis*-induced periodontitis aggravated neuroinflammation and promoted tau protein hyperphosphorylation leading to neuronal loss. The impaired meningeal lymphatic drainage and disrupted BBB affected the brain barrier function, further inhibiting the clearance of pathogenic substances and increasing the infiltration of immune cells in periodontitis mice. Brain barrier dysfunction, especially meningeal lymphatic drainage, may play an important role in linking periodontitis and cognitive impairment.

## Data Availability

The raw data supporting the conclusions of this article will be made available by the authors, without undue reservation.
